# Horizontal gaze-evoked nystagmus in pontine gaze palsy: patterns and anatomical correlates

**DOI:** 10.3389/fneur.2025.1624192

**Published:** 2025-07-10

**Authors:** Seo-Young Choi, Jae-Hwan Choi, Hyun Sung Kim, Ju-Young Lee, Sun-Uk Lee, Seung-Han Lee, Jae-Myung Kim, Hyun Ah Kim, Ji-Yun Park, Kwang-Dong Choi

**Affiliations:** ^1^Department of Neurology, Pusan National University Hospital, Pusan National University School of Medicine and Biomedical Research Institute, Busan, Republic of Korea; ^2^Department of Neurology, Pusan National University Yangsan Hospital, Pusan National University School of Medicine, Research Institute for Convergence of Biomedical Science and Technology, Yangsan, Republic of Korea; ^3^Department of Neurology, Eunpyeong St. Mary's Hospital, College of Medicine, The Catholic University of Korea, Seoul, Republic of Korea; ^4^Department of Neurology, Korea University Medical Center, Seoul, Republic of Korea; ^5^Department of Neurology, Chonnam National University Medical School, Gwangju, Republic of Korea; ^6^Department of Neurology, Keimyung University School of Medicine, Daegu, Republic of Korea; ^7^Department of Neurology, Ulsan University Hospital, University of Ulsan College of Medicine, Ulsan, Republic of Korea

**Keywords:** abducens nucleus, paramedian pontine reticular formation, gaze-evoked nystagmus, horizontal gaze palsy, pons

## Abstract

**Background:**

To delineate the patterns and anatomical correlates of gaze-evoked nystagmus (GEN) in horizontal gaze palsy due to dorsal pontine lesions.

**Methods:**

A total of 17 patients with horizontal gaze palsy and unilateral dorsal pontine lesions were retrospectively recruited from referral-based six university hospitals in Korea. The clinical characteristics, oculographic data, and MRI lesions of the patients were subjected to analysis.

**Results:**

Patients had complete (*n* = 10, 60%) or partial (*n* = 7, 40%) horizontal gaze palsy. Ten patients (60%) showed contralesional horizontal-torsional spontaneous nystagmus. Horizontal GEN was identified in 14 of the 17 patients (82%), which was contralesional (n = 8, 57%), bilateral (*n* = 5, 36%), and ipsilesional (*n* = 1, 7%). The lesion overlays revealed that damage to the surrounding area of the abducens nucleus was responsible for the generation of GEN in patients with pontine gaze palsy.

**Conclusion:**

Horizontal GEN is a common manifestation of pontine gaze palsy. In lesions in the vicinity of the abducens nucleus, the selective or extensive disruption of the connections between the ipsilateral or contralateral horizontal neural integrators and the abducens nucleus may result in diverse patterns of horizontal GEN.

## Introduction

The abducens nucleus serves as the horizontal gaze center, containing both lateral rectus motor neurons and internuclear neurons that are connected to the contralateral medial rectus through the oculomotor nucleus. This nucleus is also a hub for all neurological pathways associated with horizontal saccades, vestibulo-ocular reflex (VOR), and smooth pursuit (SP) (1, 2). Lesions affecting the abducens nucleus can lead to loss of all conjugate movements toward the side of the lesion, which is referred to as “ipsilateral horizontal gaze palsy.” The paramedian pontine reticular formation (PPRF) lesions can be clinically distinguished by selective impairment of ipsilateral saccades, while abducens nucleus lesions affect nearly all horizontal eye movements toward the lesion side (1). However, the fact that the abducens nucleus receives direct projections from the PPRF presents a challenge in differentiating between lesions of these structures.

Gaze-evoked nystagmus (GEN) represents a distinctive ocular motor dysfunction from damage to the neural integrator (1). When the eyes move from the center to an eccentric position in response to a pulse signal from the brainstem saccadic burst neurons, the step signal that sustains the final eye position against the elastic restoring forces of the extraocular muscles is generated by the neural integrator (1). An unstable neural integrator results in an inadequate sustained eye position signal, causing the eye to drift back from the eccentric position. This drift is followed by corrective saccades, known as GEN (1). The principal structures of the horizontal neural integrator in the brainstem are the medial vestibular nucleus (MVN) and the nucleus prepositus hypoglossi (NPH), which are interconnected by pathways that extend to the dorsal pons. Occasionally, anecdotal case reports have shown that lesions of the abducens nucleus or PPRF, in conjunction with conjugate horizontal gaze palsy, can result in nystagmus when gaze is directed into the intact contralateral hemifield with quick phases directed away from the lesioned side (3). However, no systematic study has been conducted to clarify this phenomenon in pontine gaze palsy.

This study aims to investigate the patterns and anatomical correlation of GEN in horizontal gaze palsy due to dorsal pontine lesions.

## Methods

### Participants

We retrospectively recruited 17 patients (11 men, median age = 69, range = 37–80) with horizontal gaze palsy due to unilateral dorsal pontine lesions from six referral centers between January 2018 and March 2022. Brain imaging was performed in all patients: 14 underwent magnetic resonance imaging (MRI), and 3 underwent cranial computed tomography (CT). Cavernous malformations (*n* = 2) and hemorrhagic lesions (*n* = 1) were identified on CT, while ischemic lesions were confirmed on MRI (Supplementary Table 1). We collected data on clinical information, oculographic, and neuroimaging results of the patients.

### Bedside neuro-otologic and neuro-ophthalmologic evaluation

Given the presence of horizontal gaze palsy, GEN was considered present if nystagmus was elicited during attempts to gaze laterally, regardless of the presence or severity of gaze palsy. In patients with spontaneous nystagmus, lateral gaze in the direction of the nystagmus was classified as GEN only if the intensity of nystagmus increased compared to central gaze. Horizontal saccades and SP were assessed within the limits imposed by the patients’ gaze palsy. The VOR was evaluated by passively rotating the head horizontally in a sinusoidal pattern at approximately 0.5 Hz, while observing the compensatory eye movements. Complete gaze palsy was defined as the inability of the eyes to move past the midline toward the side of the lesion. Partial gaze palsy was defined as a reduced but preserved ability to move beyond the midline toward the lesion side.

### Recording of 3D video-oculography

Seven patients (7/17, 41%) underwent 3-dimensional video-oculography (VOG; SLVNG, SLMED, Seoul, South Korea). VOG was not routinely performed at all participating centers, which limited its availability to 41% of the study population. VOG included assessments of spontaneous nystagmus (SN) with and without fixation, horizontal GEN, horizontal saccades, SP, and VOR. Recordings were performed with the patient seated and the head stabilized in the darkroom. A visual target positioned 1.5 meters away moved up to 30 degrees horizontally. SN was evaluated with and without fixation. Horizontal GEN was assessed using a target placed 30 degrees laterally. In the presence of gaze palsy, GEN was considered present if nystagmus appeared during attempted lateral gaze, irrespective of the gaze range limitation. Horizontal saccades were evaluated by shifting the target between ±30 degrees from center, with intermediate transitions at ±15 degrees to assess both centripetal and centrifugal movements. SP was assessed by moving the target sinusoidally at 0.5 Hz across a 30-degree horizontal range. Due to gaze palsy, the eyes could not always fully track the target; in such cases, saccades and SP were evaluated based on maximal horizontal eye movements achievable within each patient’s individual range. VOR was recorded by passively rotating the patient’s head left and right at 0.5 Hz, while monitoring the corresponding compensatory eye movements.

### Lesion analysis

Lesion analysis was performed in 14 of the 17 patients who exhibited GEN. Diffusion-weighted and T1-weighted MRI scans were spatially normalized to the Montreal Neurological Institute (MNI) template using SPM8.^1^ Lesions were manually traced on each patient’s normalized structural image using MRIcron,^2^ based on the consensus of two neurologists blinded to clinical data. For patients with left-sided lesions, the lesion masks were flipped to the right side to facilitate group-level analysis. Overlap lesion maps were generated using lesion density plots density plots (4) to visualize the regions most frequently affected among patients with GEN.

### Standard protocol approvals, registrations, and patient consents

All experiments followed the tenets of the Declaration of Helsinki and were approved by the institutional review board of Pusan National University Hospital (2003–011-088). Written informed consents were obtained after the nature and possible consequences of this study had been explained to the participants.

### Data availability

Anonymized data not published within this article will be made available by request from any qualified investigator.

## Result

### Clinical characteristics

The patients had either complete (*n* = 10, 60%) or partial (*n* = 7, 40%) horizontal gaze palsy. Ten of the 17 patients (60%) showed contralesional horizontal-torsional spontaneous nystagmus. Horizontal GEN was identified in 14 patients (82%), which was contralesional (*n* = 8, 57%), bilateral (*n* = 5, 36%), or ipsilesional (*n* = 1, 7%). In eight patients with GEN and complete gaze palsy, GEN was contralesional in seven and bilateral in one. Six patients with GEN and partial gaze palsy showed bilateral (*n* = 4), contralesional (*n* = 1), or ipsilesional GEN (*n* = 1) (Supplementary Table 1).

Eight of the 17 patients showed characteristic ocular motor findings compatible with lesions of the PPRF including conjugate selective saccadic palsy toward the side of the lesion (*n* = 4) and conjugate saccadic palsy and SP impairment with normal VOR (*n* = 4). In the intact hemifield of gaze, centripetal saccades were also markedly slow in five patients. Another three patients demonstrated distinctive findings indicative of lesions in the abducens nucleus. These included the loss of all conjugate eye movements toward the side of the lesion and normal or mild slowing of centripetal saccades in the intact hemifield of gaze. Except for two patients for whom no description of the VOR was provided, the remaining four revealed a combination of ocular motor abnormalities suggesting lesions affecting both the PPRF and abducens nucleus. They showed loss of all conjugate eye movements toward the side of the lesion and severe slowing of centripetal saccades in the intact hemifield of gaze.

### Representative cases

**Figure 1:**
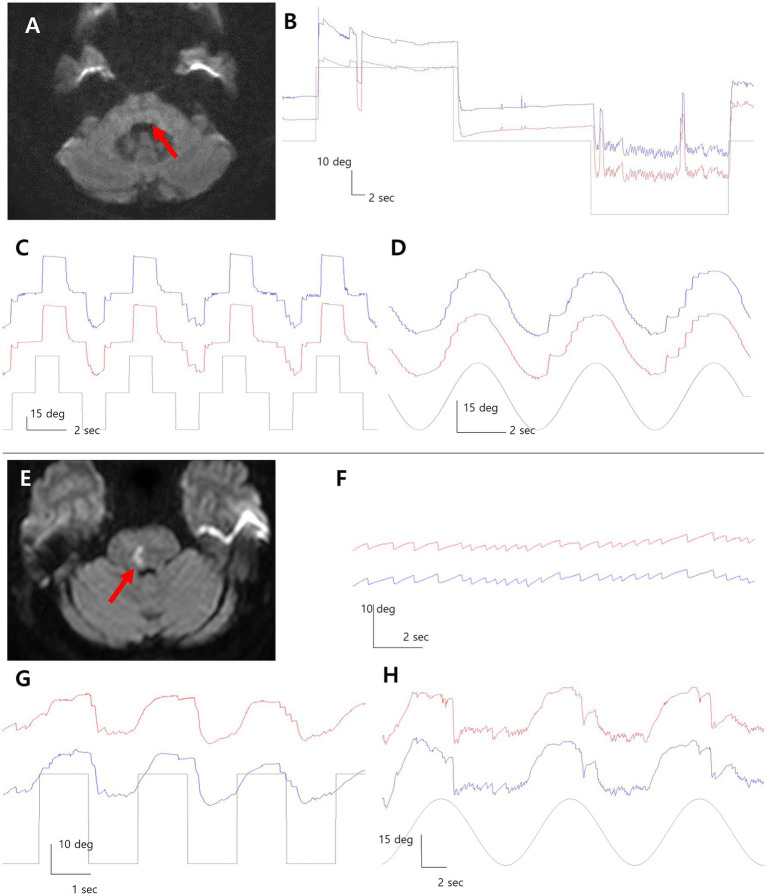
Diffusion-weighted imaging reveals focal infarcts in the dorsal pons in both patients (**A**, **E**; red arrows). In Patient 1 with left pontine infarction, video-oculography (VOG) shows left-beating gaze evoked nystagmus during leftward gaze rebound nystagmus upon return to center **(B)**. Leftward saccades are slowed, and smooth pursuit is impaired bilaterally, more on the right **(C,D)**. In Patient 2 with right pontine infarction **(E)**, left-beating GEN is observed during leftward gaze with right gaze palsy **(F)**. Horizontal saccades are restricted to the left hemifield with bilateral pursuit impairment **(G,H)**. Blue line = left eye; red line = right eye; grey line = target position.

Patient 1 was a 58-year-old man with a focal infarct in the left dorsal pons, as revealed by diffusion-weighted imaging (Figure 1A). VOG demonstrated horizontal GEN during leftward gaze, characterized by left-beating nystagmus with a mean slow-phase velocity of approximately 11°/sec and an exponentially decreasing waveform (Figure 1B). Rebound nystagmus was observed upon return to central gaze. Horizontal saccades were markedly slowed in the leftward direction (leftward velocity: 93°/sec; rightward: 197°/sec) (Figure 1C). SP was impaired in both directions, more prominently towards the right (Figure 1D).

Patient 2 was a 59-year-old woman with a right dorsal pontine infarct on diffusion-weighted imaging (Figure 1E). Leftward gaze elicited left-beating horizontal GEN on VOG, with a slow-phase velocity of approximately 6°/sec and an exponentially decreasing pattern (Figure 1F). Horizontal saccades were preserved only in the left hemifield due to right gaze palsy, with markedly slowed saccades in both directions (rightward: 22°/sec; leftward: 92°/sec) (Figure 1G). Bilateral impairment of SP was also noted (Figure 1H).

### Lesion analysis

**Figure 2:**
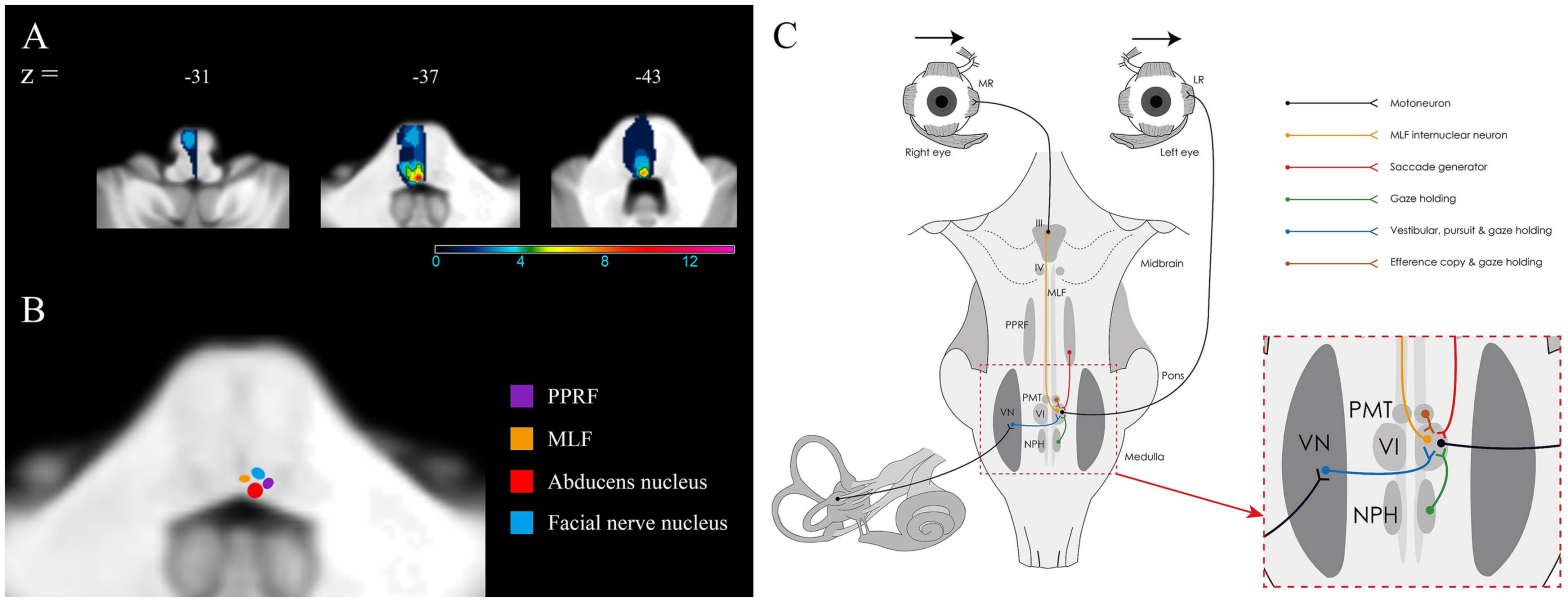
Lesion analyses in 14 patients with GEN and dorsal pontine lesions. **(A)** The surrounding area of the abducens nucleus is the most frequently involved structure (red). The numbers of overlapping lesions are illustrated by different colors from violet (*n* = 1) to red (*n* = 14). **(B)** Illustration of the areas corresponding to the abducens nucleus, paramedian pontine reticular formation, medial longitudinal fasciculus, and facial nucleus in 2 representative templates of the SUIT toolbox. **(C)** Illustration of the anatomical connections between horizontal neural integrators in the brainstem and the abducens nucleus.

All patients had unilateral dorsal pontine lesion including ischemic stroke (*n* = 13), hemorrhagic stroke (*n* = 1), cavernous malformation (*n* = 2), and multiples sclerosis (*n* = 1). One patient (patient 15) had additional bilateral infarcts in the cerebellum. Lesion overlays in 14 patients with GEN demonstrated that damage to the surrounding area of the abducens nucleus was the common area of injury (Figure 2A). Because the structures involved in horizontal gaze are clustered within the dorsal pons, it is often difficult to distinguish each component on MRI (Figure 2B). A schematic diagram of the neural pathway centered around the abducens nucleus (Figure 2C), in conjunction with oculomotor findings, may aid in the interpretation of eye movement abnormalities.

## Discussion

This study showed that horizontal GEN is a prevalent phenomenon in horizontal gaze palsy resulting from dorsal pontine lesions. Our findings are inconsistent with those of previous reports, which have revealed that lesions of the abducens nucleus or PPRF developed horizontal GEN only during contralesional gaze (1). In the present study, approximately 50% of patients showed contralesional GEN, while 36% exhibited bilateral GEN, and one patient had horizontal GEN during ipsilesional gaze only. The higher incidence of contralesional GEN in our and previous studies may be ascribed to the possibility that patients with complete saccadic palsy do not generate nystagmus during ipsilesional gaze. Indeed, our patients with complete gaze palsy mostly revealed contralesional GEN, whereas most of the patients with partial gaze palsy had bilateral or ipsilesional GEN.

The abducens nucleus receives projections from neurons within the MVN and NPH that serve as the horizontal neural integrators (1). A selective or extensive disruption of the connections between the ipsilateral or contralateral horizontal neural integrators and the abducens nucleus may result in different patterns of horizontal GEN in lesions affecting the vicinity of the abducens nucleus. The pathways between the MVN and abducens nucleus are ipsilateral for the inhibitory fibers and contralateral for the excitatory fibers, the latter passing close proximity to and through the ipsilateral abducens nucleus before crossing the midline. In the lesion surrounding the abducens nucleus, damage to the contralateral excitatory fibers originating from both MVNs may result in contralesional or ipsilesional GEN, or both. Actually, an isolated lesion of the MVN results in contralesional horizontal-torsional spontaneous nystagmus and horizontal GEN during contralesional gaze, which is the most commonly observed pattern in our patients (5). This finding indicates an interruption of the contralesional excitatory fibers from the ipsilesional MVN.

Bilateral horizontal GEN in pontine gaze palsy can be also attributed to the damage to the NPH, which is located in close proximity to the abducens nucleus, or to the fibers from the NPH to the ipsilateral abducens nucleus in the lesion surrounding the abducens nucleus (6). Given that the NPH connects bilaterally through the inferior olivary nucleus to the flocculus, damage to this pathway can induce hypofunction of the ipsilesional inferior olivary nucleus and disinhibition of the contralateral flocculus (6, 7). A unilateral lesion involving the NPH developed ipsilesional spontaneous nystagmus with asymmetric horizontal GEN that is greater during ipsilesional gaze (8).

An additional possibility is damage to a subset of cell groups of the paramedian tracts (PMT), which is located on the rostral cap of the abducens nucleus and contains floccular-projection neurons that may play a role in gaze-holding (9). Selective inactivation of the PMT in monkey results in GEN with exponentially decreasing slow phases in the horizontal and vertical directions (10). Given that the PMT-floccular pathway may be involved in an intrinsic mechanism of the integrator that sets the neutral eye position, it can be postulated that GEN observed during gaze to the side opposite the lesion may result from a pathologically recalculated neutral eye position (10). Although the boundaries of the lesion are unclear, a case of infarction involving the PMT with omnidirectional GEN has been reported (11). The contralesional horizontal GEN in our and previous studies can be explained by the damage to the PMT. However, the absence of GEN in the vertical plane is not compatible with this assumption.

This study has several limitations. First, because the participants had gaze palsy, accurate calibration of VOG device may not have been feasible. As a result, we did not report quantitative measurements of eye position or velocity in the manuscript. Instead, we verified the presence of ocular motor abnormalities by reviewing the VOG recordings in conjunction with the corresponding video images. Second, most ocular motor evaluations were performed at the bedside. Since VOG was not routinely conducted at all participating centers, VOG data were available for only 7 patients, and we were unable to quantify the findings across the entire cohort. Future prospective studies may be necessary to address this limitation. Third, because the lesions in patients with cavernous malformation or hemorrhage were confirmed only by CT, these patients were excluded from lesion-based analysis using MRI. Lastly, among the five patients who exhibited contralesional spontaneous nystagmus, the presence of contralesional GEN during contralesional gaze could potentially be misinterpreted as an enhancement of the spontaneous nystagmus due to Alexander’s law, rather than true GEN. In future studies, detailed analyses of the slow-phase velocity using VOG and assessments for subtle GEN during ipsilesional gaze may help to differentiate these phenomena more clearly.

## Conclusion

Horizontal GEN is a frequent and variable finding in pontine gaze palsy. Lesions near the abducens nucleus appear critical for GEN generation, likely due to disruption of horizontal neural integrator pathways. These results underscore the anatomical basis of GEN and support its diagnostic value in dorsal pontine lesions.

## Data Availability

The original contributions presented in the study are included in the article/Supplementary material, further inquiries can be directed to the corresponding author.

## Footnotes

^1^ www.fil.ion.ucl.ac.uk/spm/software/spm8

^2^ www.mccauslandcenter.sc.edu/mricro/mricron
