# Supplementary material for: Horizontal gaze-evoked nystagmus in pontine gaze palsy: patterns and anatomical correlates
Source: Front Neurol. 2025 Jul 10;16:1624192. doi: 10.3389/fneur.2025.1624192 (PMC12286784; doi:10.3389/fneur.2025.1624192)
Supplement: Supplementary file 1 [file Table_1.docx]

**Supplementary Table.** Clinical characteristics and abnormal ocular motor findings in the patients.

| **Patients no.** | **Age** | **Sex** | **Etiology** | **Lesion side** | **Severity of gaze palsy** | **SN** | **GEN** | **Saccades from contralateral field to center** | **Smooth pursuit** | **VOR** | **Accompanying signs** |
| --- | --- | --- | --- | --- | --- | --- | --- | --- | --- | --- | --- |
| 1 | 69 | M | Infarction | L | Partial | (-) | I | Mild slowing | Impaired | Impaired | (-) |
| 2 | 59 | F | Infarction | R | Partial | (-) | C | Severe slowing | Intact | Intact | L hemiparesis, dysarthria |
| 3 | 79 | M | Infarction | L | Partial | (-) | (-) | Mild slowing | Impaired | Intact | (-) |
| 4 | 80 | M | Infarction | L | Complete | C | C | Severe slowing | Intact | Intact | (-) |
| 5 | 58 | M | Infarction | L | Complete | C | C | Mild slowing | Impaired | Impaired | (-) |
| 6 | 78 | F | Infarction | R | Complete | C | C | Severe slowing | Impaired | Intact | (-) |
| 7 | 69 | F | Infarction | R | Complete | C | (-) | Mild slowing | Impaired | Impaired | R pph. facial palsy |
| 8 | 56 | M | Cavernous malformation | L | Complete | (-) | (-) | Severe slowing | Intact | Intact | (-) |
| 9 | 65 | F | Infarction | R | Partial | (-) | B | Mild slowing | Impaired | Intact | L ataxia, |
| 10 | 72 | F | Infarction | R | Complete | C | C | Severe slowing | Impaired | Impaired | L sensory change and dysarthria |
| 11 | 64 | M | Infarction | R | Partial | C | B | Mild slowing | Impaired | ND | R pph. facial palsy |
| 12 | 77 | M | Hemorrhage | R | Complete | (-) | C | Severe slowing | Impaired | Impaired | R pph. facial palsy |
| 13 | 75 | M | Infarction | L | Complete | (-) | C | Severe slowing | Intact | Intact | (-) |
| 14 | 37 | M | Multiple sclerosis | L | Complete | C | C | Severe slowing | Impaired | Impaired | B ataxia, facial palsy |
| 15 | 70 | M | Infarction | R | Complete | C | B | Severe slowing | Impaired | Impaired | R Horner’s syndrome,  R ataxia |
| 16 | 56 | F | Cavernous malformation | L | Partial | C | B | Mild slowing | Impaired | Intact | L ataxia, L Horner’s syndrome |
| 17 | 43 | M | Infarction | L | Partial | C | B | Mild slowing | Impaired | ND | L Horner’s syndrome, dysarthria, L ataxia |

L=left; R=right; SN=spontaneous nystagmus; C=contralesional; GEN=gaze-evoked nystagmus; I=ipsilesional; B=bilateral; VOR=vestibulo-ocular reflex; ND=not done; pph.=peripheral
